# Antioxidant-Rich Extract from Plantaginis Semen Ameliorates Diabetic Retinal Injury in a Streptozotocin-Induced Diabetic Rat Model

**DOI:** 10.3390/nu8090572

**Published:** 2016-09-18

**Authors:** Thing-Fong Tzeng, Wayne Young Liu, Shorong-Shii Liou, Tang-Yao Hong, I-Min Liu

**Affiliations:** 1 Department of Pharmacy and Master Program, Collage of Pharmacy and Health Care, Tajen University, Pingtung County 90741, Taiwan; d850084@yahoo.com.tw (T.-F.T.); ssliou@tajen.edu.tw (S.-S.L.); 2 Department of Urology, Jen-Ai Hospital, Taichung City 41625, Taiwan; waynedoctor@gmail.com; 3 Center for Basic Medical Science, Collage of Health Science, Central Taiwan University of Science and Technology, Taichung City 40601, Taiwan; 4 Department of Biotechnology, Collage of Pharmacy and Health Care, Tajen University, Pingtung County 90741, Taiwan; tyhong@tajen.edu.tw

**Keywords:** plantaginis semen, diabetic retinopathy, STZ-diabetic rats, phenolic antioxidants extract

## Abstract

Plantaginis semen, the dried ripe seed of *Plantago asiatica* L. or *Plantago depressa* Willd. (Plantaginaceae), has been traditionally used to treat blurred vision in Asia. The aim of this work was to investigate the effect of plantaginis semen ethanol extract (PSEE) on the amelioration of diabetic retinopathy (DR) in streptozotocin (STZ)-diabetic rats. PSEE has abundant polyphenols with strong antioxidant activity. PSEE (100, 200 or 300 mg/kg) was oral administrated to the diabetic rats once daily consecutively for 8 weeks. Oral administration of PSEE resulted in significant reduction of hyperglycemia, the diameter of the retinal vessels, and retinal vascular permeability and leukostasis in diabetic rats. In addition, PSEE administration increased the activities of superoxidase dismutase (SOD) and catalase (CAT), and glutathione peroxidase (GSH) level in diabetic retinae. PSEE treatment inhibited the expression of vascular endothelial growth factor (VEGF) and hypoxia-inducible factor-1α (HIF-1α) and the phosphorylation of Akt without altering the Akt protein expression in diabetic retinae. PSEE not only down-regulated the gene expression of hypoxia-inducible factor-1α (TNF-α) and interleukin-1β (IL-1β), but also reduced ICAM-1 and VCAM-1 expression in diabetic retinae. Moreover, PSEE reduced the nuclear factor-κB (NF-κB) activation and corrected imbalance between histone deacetylases (HDAC) and histone acetyltransferases (HAT) activities in diabetic retinae. In conclusion, phenolic antioxidants extract from plantaginis semen has potential benefits in the prevention and/or progression of DR.

## 1. Introduction

Diabetes mellitus (DM) is a severe metabolic disease, and numerous complications are associated with the characteristic hypergly-cemia of this disease [1]. Of these, diabetic retinopathy (DR) is one of the major microvascular complications amongst diabetic patients, and is the primary cause of visual loss [1]. Increasing evidence indicates that the chronic uncontrolled hyperglycemic state leads to generation of reactive oxygen species (ROS), which triggers a severe inflammatory state characterized by an elevation of proinflammatory cytokines [2,3,4]. The proinflammatory cytokines tumor necrosis factor-α (TNF-α) and interleukin-1β (IL-1β) are positively correlated with blood retinal barrier (BRB) breakdown and vascular cell death [5,6,7]. Apart from this, chronic hyperglycemia stimulates synthesis and secretion of vascular endothelial growth factor (VEGF), which was transcriptional regulated by hypoxia-inducible factor-1α (HIF-1α), is the major growth factor mediating retinal vascular leakage and neovascularization [8,9].

There is growing evidence that the number of patients with DR keeps growing due to difficulty in achieving tight glycemic control, unresponsive to the current therapeutic approaches and significant side-effects from therapies [10]. Laser photocoagulation is currently the primary method of treatment for patients with diabetic retinopathy who are at a high risk of vision loss, but unfortunately this is not always effective for improving vision [11]. There is a great need to develop new therapeutic approaches for this devastating disease. In recent years, clinical and epidemiological evidence suggests that a diet rich in polyphenol may decrease the risk of chronic diseases associated with oxidative stress including diabetes [12,13].

Plantaginis semen (Cheqianzi), the dried, ripe seed of *Plantago* species (Plantaginaceae), has been traditionally used as medicines and supplements for improving blurred vision or internal oculopathy due to yin deficiency of the liver and kidneys in Asia [14]. *Plantago asiatica* L. and *Plantago depressa* Willd are the official sources of plantaginis semen in Chinese Pharmacopoeia [15]. These medicinal materials are produced in all parts of China, collected in summer and autumn when the seed is ripe [15]. Plantaginis semen has other uses in folk medicine including treating edema, dysuria, stranguria with burning pain and blood, and removing dampness to arrest diarrhea as well as clearing heat from the lungs and resolve phlegm [16,17]. One of the major constituents in plantaginis semen is mucilage, which has activity to lower glycemic index of food in human [18]. In addition, flavonoids are abundant in plantaginis semen [19]. It is apparent that the polyphenols could improve glucose homeostasis through potential multiple mechanisms of action [13]. The evidence also suggested that polyphenolic flavonoids are capable of acting on various mechanisms or etiological factors responsible for the development of different sight threatening ocular diseases [20]. Thus, plantaginis semen seems be valued for adjuvant therapy in the control of free radical-mediated diseases and/or diabetes-related microvascular complications. However, there are no scientifically proven data to show plantaginis semen is responsible for the protective effect on diabetic retinal tissues.

STZ-induced type 1 diabetes in rodents are commonly used as DR animal model. Many researches used STZ-diabetic rats to study DR and other diabetic complications [21]. The goal of this study was to determine whether plantaginis semen can be used for treatment of STZ-induced DR in rats and its underlying mechanism.

## 2. Materials and Methods

### 2.1. Preparation of Plant Extracts

Plantaginis semen were purchased from Jinbaoan Trade Co., Ltd. (Zhunan Township, Miaoli County, Taiwan) in September 2015, and identified by Hong T.Y. (Department of Biotechnology, Collage of Pharmacy and Health Care, Tajen University, Taiwan). The voucher specimen (Lot No. PS 20150926) was deposited in our laboratory. Plantaginis semen (10 kg) were grounded into a 40-mesh powder and extracted with 95% ethanol (5 volumes of ethanol) in a stainless steel extraction tank for 24 h at room temperature. This ethanol mixture was filtered through funnels and centrifuged (4 °C, 1350× *g*, 20 min), and this was repeated three times. All of the precipitate was eliminated, and the ethanol extract portion was collected and concentrated using a rotary evaporator. Plantaginis semen ethanol extract (PSEE) was then evaporated under reduced pressure conditions, which completely eliminated the alcohol, followed by lyophilization, yielding approximately 1328 g of dry residue (*w*/*w* yield: 13.2%). PSEE was kept at −20 °C until use and suspended in distilled water.

### 2.2. Total Phenolic Content

Polyphenol content of PSEE was determined according to the Folin-Ciocalteu colorimetric method [22]. Standard gallic acid (Sigma-Aldrich, Inc., Saint Louis, MO, USA) and an aliquot of PSEE were diluted with an ethanol/water (60:40, *v*/*v*) solution containing 0.3% HCl. Two mL of 2% Na_2_CO_3_ was mixed into each sample of 100 μL and allowed to equilibrate for 2 min before adding 50% Folin-Ciocalteu’s phenol reagent (Sigma-Aldrich, Inc.). Absorbance at 750 nm was measured at room temperature. The polyphenol content of PSEE was expressed as mg of gallic acid equivalent per gram (g) of PSEE in dry weight (DW), i.e., mg gallic acid/g DW.

### 2.3. Total Flavonoid Content

The total flavonoid content of PSEE was determined by the aluminium chloride colorimetric method [23]. Briefly, 0.25 mL of PSEE (100 μg/mL) was added to a tube containing 1 mL of double-distilled water. Next, 0.075 mL of 5% NaNO_2_, 0.075 mL of 10% AlCl_3_, and 0.5 mL of 1 mmol/L NaOH were added sequentially at 0, 5, and 6 min. Finally, the volume of the reacting solution was adjusted to 2.5 mL with double-distilled water. The solution had an absorbance of 510 nm. The total flavonoid content was calculated from a calibration curve, and the result was expressed as mg rutin (Sigma-Aldrich, Inc.) equivalent per g dry weight, i.e., mg rutin/g DW.

### 2.4. Total Antioxidant Capacity

The total antioxidant capacity of PSEE was determined using the horseradish peroxidase catalyzed oxidation of 2,2-azino-bis-(3-ethyl benzothiazoline-6-sulfonicacid) (ABTS) [24]. The reaction mixture contained 0.5 mL of 1000 μmol/L ABTS (in ddH_2_O) and 3.5 mL of 100 μmol/L H_2_O_2_. The reaction was started by adding 0.5 mL of 44 U/mL peroxidase (in 0.1 mol/L phosphate-buffered saline (PBS)). After 1 h, 0.05 mL of PSEE were added to the mixture. After 5 min, absorbance was measured at 730 nm. Trolox (Sigma-Aldrich, Inc.) standard solution were prepared and assayed under the same conditions. Results were expressed in terms of trolox equivalent antioxidant capacity (TEAC), i.e., mmol/L Trolox/100 g DW.

### 2.5. Experimental Animals

All experimental methods and animal care procedures were approved by the Institutional Animal Care and Use Committee (IACUC) of Tajen University (approval number, IACUC 104-28; approval date: 12 November, 2015), in accordance with the Guide for the Care and Use of Laboratory Animals of the National Institutes of Health, as well as the guidelines of the Animal Welfare Act. Male Wistar rats (8 weeks aged) weighting 200–250 g, were purchased from National Laboratory Animal Center (Taipei, Taiwan) and housed two per cage in a room under controlled temperature (20–25 °C), humidity (50% ± 5%) and lighting (12 h light/dark cycle) with food and water provided ad libitum. Rats were rendered diabetic by a single intravenous injection of 60 mg/kg streptozotocin (STZ; Sigma-Aldrich, Inc.). Eight-week age-matched control rats were injected with vehicle (sterile saline 0.9%, pH 7.4). After 1 week, rat with non-fasting blood glucose levels >350 mg/dL, polyuria, and glucosuria were defined as diabetic and used for the experiments.

### 2.6. Treatment Protocols

In the treatment group (𝑛 = 10 per group), STZ-diabetic rats were dosed by oral gavage once per day for 8 weeks with PSEE at dosages of 100, 200, or 300 mg/kg in a volume of 1.5 mL/kg distilled water. The dosages of PSEE were selected based on consideration of tests of Plantago extracts relating to producing preventive effects on oxidative damage in rats [25]. A vehicle-treated group (𝑛 = 10 per group). Of normal rats and STZ-diabetic rats were treated with 1.5 mL/kg distilled water only over the same treatment period. Animals had free access to standard rat diet (Harlan Teklad, Madison, WI, USA; catalogue number (Cat. No.) 2018) and water throughout the entire treatment period.

At the end of the 8-week treatment, the rats were weighed, fasted overnight and anesthetized using an intraperitoneal injection of sodium pentobarbital (60 mg/kg). While under anesthesia, they were painlessly sacrificed and blood was collected from the abdominal aorta of each animal into heparin sample bottles. Rat eyes from each group were removed and the retinae were isolated. The diagnostic kit for determination for plasma levels of glucose (Cat. No. COD12503) was purchased from BioSystem (Barcelona, Spain). Commercial enzyme-linked immunosorbent assay (ELISA) kits were used to quantify HbA_1c_ levels (Integrated Bio Ltd., Taipei, Taiwan; Cat. No. CSB-E08140r). All analyses were performed in accordance with the instructions provided by the manufacturers.

### 2.7. Fundus Photography and Vessel Diameter

Fundus photography is performed with a retina camera (Kowa Company Ltd., Tokyo, Japan). In order to accustom to the fundus photography procedure, rats were trained before start of the study. Eyes were dilated with a drop of 1% tropicamide (Synpac-Kingdom Pharmaceutical Co., Ltd., Taipei, Taiwan). Moisol eye drops were administered periodically to prevent the cornea from drying out. Fundus photography was done regularly till 8 weeks to monitor the fundus changes.

The diameter of retinal vessels was estimated by previously described method [26]. Before diameter estimation, the retinal photographs from all groups were randomized. The vessel diameter of 3 most prominent vessels was estimated at 3 sites in its widest portion at equal distance from the center. An average of 3 estimations was taken as the final retinal vessel diameter.

### 2.8. Quantification of Retinal Leukostasis

Quantification of leukostasis was performed at the end of the 8-week treatment by previously described method [27]. The chest cavity of each deeply anesthetized rat was carefully opened and a perfusion needle was inserted into the left ventricle. After cutting the right atrium, the animals were immediately perfused with 500 mL of PBS per kg body weight and heparin (0.1 mg/mL) to wash out nonadherent blood cells. Fluorescein isothiocyanate-coupled Concanavalin A lectin (ConA) (20 μg/mL in PBS; pH 7.4; 5 mg/kg; Vector Laboratories, Burlingame, CA, USA) was then perfused to label adherent leukocytes and vascular endothelial cells. Residual unbound ConA was flushed by PBS perfusion. Eyes were removed and fixed in 4% paraformaldehyde for 1 h. Retinas were dissected and flat mounted on a microscope slide, covered with anti-fading medium and a coverslip, and imaged via fluorescence microscopy. Only whole retinae in which the entire vascular network was stained were used for analysis. The total number of adherent leukocytes within the vessels of each retina was counted.

### 2.9. Retinal Permeability Assessment

Retinal vascular permeability was measured using Evans blue (EB) dye extravasation technique at the end of the 8-week treatment [28]. EB dye (Sigma-Aldrich, Inc.) was dissolved in normal saline at 45 mg/mL and was injected through the tail vein of anesthetized rats over 10 s at a dosage of 45 mg/kg. After the dye had circulated for 2 h, the rats were anesthetized with sodium pentobarbital (40 mg/kg), the chest cavity was opened, and cardiac perfusion was performed via the left ventricle with 1% paraformaldehyde in citrate buffer (0.05 mol/L, pH 3.5) under a constant pressure of 120 mmHg. Immediately after perfusion, the retinas were carefully dissected under an operating microscope. After retinas were fully dried at 4 °C, then the weights of them were measured, EB dye was extracted by incubating each sample in 150 µL formamide for 18 h at 70 °C. The extract was ultracentrifuged at a speed of 14,000 rpm for 60 min. Absorbance was measured using 100 µL of the supernatant at 620 nm and 740 nm. The concentration of EB in the extracts was calculated from a standard curve and normalized by total protein concentration in the tissue.

### 2.10. Assay of Retinal Antioxidant Enzymes

Retinas from right and left eyes from one rat were pooled as one sample, and then were homogenized in 10 volume of ice cold 0.1 M Tris-HCl, pH 7.4 containing 0.5% Triton X-100, 5 mmol/L β-mercaptoethanol, 0.1 mg/mL phenylmethylsulfonyl fluoride and centrifuged at 14,000× *g* for 5 min at 4 °C. The supernatant was collected and used for following experiments as described below. Protein concentration of the supernatant was assayed by Bio-Rad protein assay kit (Bio-Rad Laboratories, Hercules, CA, USA). The intracellular activity of superoxide dismutase (SOD; Cat. No. ab65354), catalase (CAT; Cat. No. ab118184) and glutathione (GSH; Cat. No. ab65322) were estimated using commercially available assay kits from Abcam plc. (Cambridge, MA, USA). All assays were carried out in triplicates.

### 2.11. Protein Extraction and Western Blot Analyses

Retinas from right and left eyes from one rat were pooled as one sample, which were then homogenized in 1 mL of ice-cold hypotonic buffer A (10 mmol/L of HEPES, 10 mmol/L of KCl, 2 mmol/L of MgCl_2_, 1 mmol/L of dithiothreitol, 0.1 mmol/L of EDTA, and 0.1 mmol/L of phenylmethylsulfonylfluoride; pH 7.8). A solution of 80 μL of 10% Nonidet P-40 was added to the homogenates, and the mixture was centrifuged for 2 min at 14,000× *g* at 4 °C. Before immunoblotting, and the protein concentration of each sample was determined using a Bio-Rad protein assay kit and bovine serum albumin as a standard, to ensure equal loading among lanes.

The tissue lysates containing 40–50 mg protein were electrophoresed through 8%, 12%, and 15% sodium dodecyl sulfate-polyacrylamide gels. According to the manufacturer’s instructions, separated proteins were electrophoretically transferred to a nitrocellulose membrane, blocked with 5% skim milk solution for 1 h, and incubated with primary antibodies to TNF-α (Cat. No. sc-1348), IL-1β (Cat. No. sc-7884), intercellular adhesion molecule 1 (ICAM-1; Cat. No. sc-8439), vascular cell adhesion molecule 1 (VCAM-1; Cat. No. sc-8304), HIF-1α (Cat. No. sc-1836), VEGF (Cat. No. sc-1836), Akt (Cat. No.sc-5298), pAkt (Ser 473) (Cat. No. sc-135651), pAkt (Thr 308) (Cat. No. sc- sc-16646-R) and β-actin (Cat. No. sc-20357) at 4 °C overnight, respectively. All antibodies were purchased from Santa Cruz Biotechnology, Inc. (Santa Cruz, CA, USA) and used at a dilution of 1:1000. After three 5 min washes in Tris-buffered saline with Tween(TBST; 20 mmol/L Tris-HCl, pH 7.5, 150 mmol/L NaCl, and 0.05% Tween 20), membranes were incubated with the appropriate peroxidase-conjugated secondary antibodies. With the ECL advance Western Blotting detection kit (Cat. No. RPN2135; GE Healthcare Life Sciences, Piscataway, NJ, USA), the membranes were washed three times in TBST and visualized on X-ray film. Band densities were determined using ATTO Densitograph Software (ATTO Corporation, Tokyo, Japan) and quantified as the ratio to β-actin. The mean value for samples was adjusted to a value of 1.0 from the vehicle-treated normal rats on each immunoblot, expressed in densitometry units. Then, all experimental sample values were expressed to this adjusted mean value.

### 2.12. Real-Time Polymerase Chain Reaction (PCR)

Total RNA was extracted from rat retinas using Trizol reagent (Invitrogen; Boston, MA, USA) according to the manufacturer’s protocol. Two retinas from right and left eyes from one rat were pooled as one sample. RNA was quantified by measuring absorbance at 260 nm and its integrity verified by agarose gel electrophoresis using ethidium bromide for visualization. For the reverse transcriptase reaction, 1 μg of total RNA per sample and 8.5 μg/μL random hexamer primers were heated at 65 °C for 5 min and then quenched on ice. This mixture was combined with 500 μmol/L each of dATP, dTTP, dCTP, and dGTP, 10 mmol/L dithiothreitol, 20 mmol/L Tris-HCl (pH 8.4), 50 mmol/L KCl, 5 mmol/L MgCl_2_, 40 units of RNaseOUTTM recombinant ribonuclease inhibitor (Invitrogen) and 100 units SuperScript III reverse transcriptase (Invitrogen). Samples were subjected to DNase (Promega; Madison, WI, USA) treatment at 37 °C for 20 min in a GeneAmp 9700 Thermal Cycler (Applied Biosystems; Foster City, CA, USA) and then held at 4 °C. After aliquots were taken for immediate use in PCR, the remainder of the cDNA was stored at −20 °C. mRNA expression was measured by quantitative real-time PCR in a fluorescent temperature Lightcycler 480 (Roche Diagnostics; Mannheim, Germany). The sequences of the primers were as follows: for TNF-α, 5′-ACACCATGAGCACGGAAAGC-3′ (forward) and 5′-CCGCCACGAGCAGGAA-3′ (reverse); for IL-1β, 5′-AATGGACAGAACATAAGCCAACA-3′ (forward) and 5′-CCCAAGGCCACAGGGAT-3′ (reverse); for ICAM-1, 5′-CGGGTTTGGGCTTCTCC-3′ (forward) and 5′-GCCACTGCTCGTCCACATAG-3′ (reverse); for VCAM-1, 5′-ATCTTCGGAGCCTCAACGG-3′ (forward) and 5′-CCAATCTGAGCGAGCGTTT-3′ (reverse); for HIF-1α, 5′-GTCGGACAGCCTCACCAAACAG-3′ (forward) and 5′-TAGGTAGTGAGCCACCAGTCATCCAAGGAA-3′ (reverse); for VEGF, 5′-ACAGGGAAGACAATGGGATGA-3′ (forward) and 5′-GGGCCAGGGATGGGTTT-3′ (reverse); for β-actin, 5′-TGTGATGGTGGGAATGGGTCAG-3′ (forward) and 5′-TTTGATGTCACGCACGATTTCC-3′ (reverse). Primers were designed using Primer Express Software version 2.0 System (Applied Biosystems; Foster City, CA, USA). The PCR reaction was performed using the following cycling protocol: 95 °C for 5 min, followed by 45 cycles of 95 °C for 5 s, 58 °C for 15 s, and 72 °C for 20 s. Dissociation curves were run after amplification to identify the specific PCR products. The mRNA expression levels were normalized to β-actin mRNA levels and calculated according to the delta-delta Ct method [29].

### 2.13. Activity of NF-κB, Histone Deacetylases (HDAC) and Histone Acetyltransferases (HAT)

Nuclear extract of retina was prepared using the nuclear extract kit (Cat. No. 40410; Active Motif, Carlsbad, CA, USA) following manufacturer's protocol. Two retinas from right and left eyes from one rat were pooled as one sample. Nuclear factor-κB (NF-κB) activation was determined, TransAM^®^ NF-κB p65 transcription factor assay kit (Cat. No. 40596) implemented under the procedures provided by the manufacturer (Active Motif Inc., Carlsbad, CA, USA). Reaction was quantified at 450 nm. Histone deacetylases (HDAC) activity in retinal nuclear extracts was measured by HDAC activity colorimetric assay kit (Cat. No. K331-100) from BioVision Inc. (Milpitas, CA, USA). The colorimetric readings were measured at 400 nm in a spectrophotometer. Activity of histone acetyltransferases (HAT) was quantified in retinal nuclear fraction by non-radioactive indirect ELISA kit (Cat. No. K332-100) from BioVision Inc. The readings were monitored at 440 nm in a spectrophotometer.

### 2.14. Statistical Analysis

All statistical analyses were performed using SPSS for Windows (version 21.0; IBM Corporation, Armonk, NY, USA). The results are presented as the mean ± standard deviation (SD) for each group of animals at the number (*n*) indicated. The significance of differences between groups was evaluated by oneway ANOVA with Fisher’s Least Significant Difference post hoc test; and *p* < 0.05 was considered as indicating statistically significant differences. Relationships between variables were examined using Pearson correlations.

## 3. Results

### 3.1. Polyphenolic Composition and Antioxidant Activity

The content of total polyphenols and flavonoids in PSEE were 184.5 ± 4.7 mg gallic acid/g DW and 63.8 ± 0.60 mg rutin/g DW, respectively. The TEAC value of PSEE was 18.3 ± 2.6 mmol/L Trolox/100 g DW.

### 3.2. Effects on Body Weights and Plasma Parameters

At the end of the experimental period, plasma levels of glucose and HbA_1c_ in STZ-treated rats were significantly greater than those in the normal animals, while body weight of the diabetic rats was significantly less than that of the normal group. Treatment 300 mg/kg/day PSEE for 8 weeks decreased the plasma levels of glucose and HbA_1c_ in STZ-diabetic rats by 33.1% and 27.9%, respectively, relative to the values in vehicle-treated counterparts (Table 1). Comparison to the vehicle-treated group, the reduction in body weight was not obvious in STZ-diabetic rats receiving PSEE at the end of experimental period (Table 1).

### 3.3. Effects on Antioxidant Parameters in Retinae

The activities of SOD and CAT in in diabetic retinae were significantly reduced as compared to those from normal group. Enhancement of SOD and CAT activities were observed in PSEE-treated 8-week diabetic retina with a dose-dependent manner (Table 1). Retinal GSH levels were markedly lower in STZ-diabetic rats compared with normal rats. Administration of STZ-diabetic rats with PSEE for 8 weeks resulted in a dose-dependent increase of GSH levels (Table 1).

### 3.4. Fundus Photographs and Microvasculature Diameter

**Figure 1:**
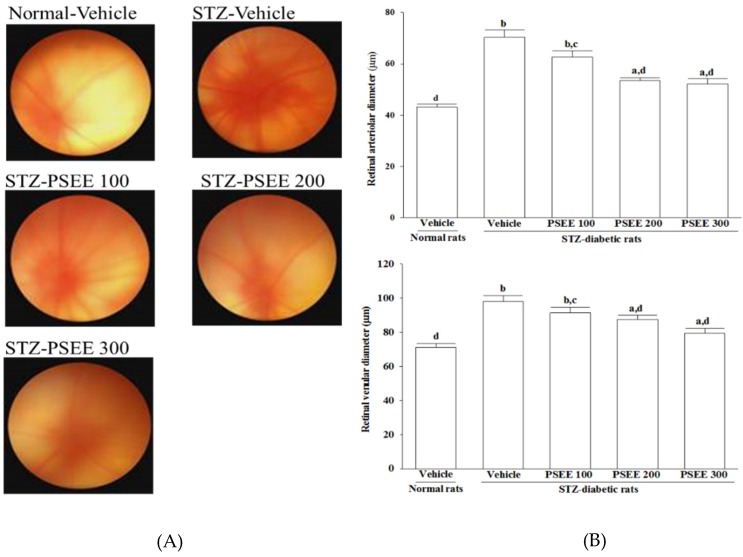
Fundus photographs (**A**) and retinal vascular diameters (**B**) in rats receiving 8-week treatments. STZ-diabetic rats (STZ) were dosed by oral gavage once per day for 8 weeks with plantaginis semen ethanol extract (PSEE) at dosages of 100 (PSEE 100), 200 (PSEE 200), or 300 mg/kg (PSEE 300). Normal or STZ-diabetic rats receiving vehicle treatment were given the same volume of vehicle (distilled water) used to prepare the test medication solutions. Fundus photograph from normal rat not showed any vascular dysfunction. Severse vascular leakage and dilatation vessels have been shown in fundus image from STZ-diabetic rats. Fundus photographs of PSEE-treated STZ-diabetic rats showed lesser dilated vessels. Values (mean ± SD) were obtained for each group of ten animals. ^a^ *p* < 0.05 and ^b^ *p* < 0.01 compared to the values of vehicle-treated normal rats, respectively. ^c^ *p* < 0.05 and ^d^ *p* < 0.01 compared to the values of vehicle-treated STZ-diabetic rats, respectively.

Fundus photograph from normal rat was not showing any abnormal in retinal vasculature and the optic nerve head (Figure 1) However, vascular leakage with accompanying dilatation vessels were shown on fundus photographs from STZ-diabetic rats (Figure 1). Less vascular leakage was present from the optic disc of STZ-diabetic rats receiving 300 mg/kg/day PSEE treatment (Figure 1). In addition, the retinal arterioles and venules became mildly dilated in PSEE (300 mg/kg/day)-treated STZ-diabetic rats (Figure 1).

### 3.5. Effects on Retinal Vascular Permeability and Leukostasis

**Figure 2:**
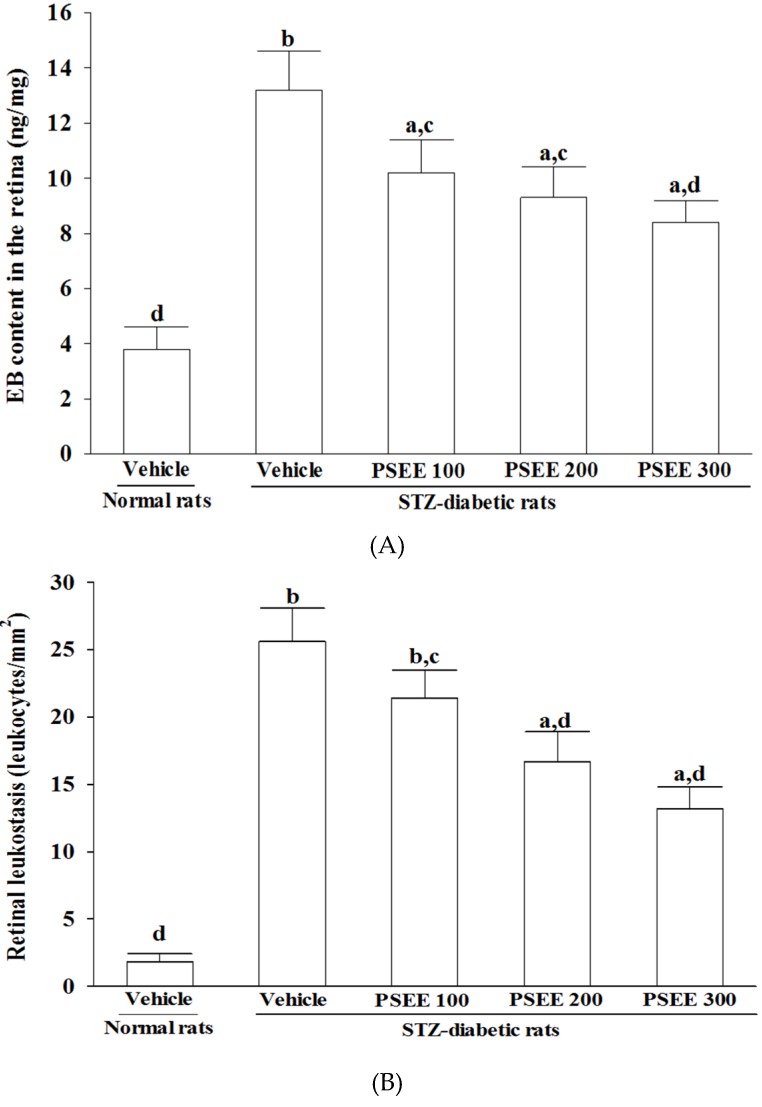
Retinal vascular permeability (**A**) and leukostasis (**B**) in rats receiving 8-week treatments. STZ-diabetic rats (STZ) were dosed by oral gavage once per day for 8 weeks with PSEE at dosages of 100 (PSEE 100), 200 (PSEE 200), or 300 mg/kg (PSEE 300). Normal or STZ-diabetic rats receiving vehicle treatment were given the same volume of vehicle (distilled water) used to prepare the test medication solutions. Retinal vascular permeability was measured with EB dye as a tracer. Evans blue was normalized by total protein concentration in the tissue. Values (mean ± SD) were obtained for each group of ten animals. ^a^ *p* < 0.05 and ^b^ *p* < 0.01 compared to the values of vehicle-treated normal rats, respectively. ^c^ *p* < 0.05 and ^d^ *p* < 0.01 compared to the values of vehicle-treated STZ-diabetic rats, respectively.

Increased leakages of EB dye were observed in the retinas of STZ-diabetic rats (Figure 2A). Treatment of STZ-diabetic rats with 100, 200 or 300 mg/kg/day PSEE for 8 weeks decreased retinal EB dye accumulation by 21.7%, 29.5% and 36.4%, respectively, when compared with the levels observed in the vehicle-paired counterparts (Figure 2A).

The number of adherent leukocytes in the retinal microvasculature of normal rats was negligible, while STZ-induced diabetes caused a significant increase of leukocytes adhesion to the endothelia cells (Figure 2B). Eight-week administration of STZ-diabetic rats with 100, 200 or 300 mg/kg/day PSEE decreased retinal leukostasis by 16.4%, 34.8%, 48.4%, respectively, compared to vehicle-treated diabetic group (Figure 2B).

### 3.6. Effects on Retinal Inflammatory Cytokines and Chemokines Expression

**Figure 3:**
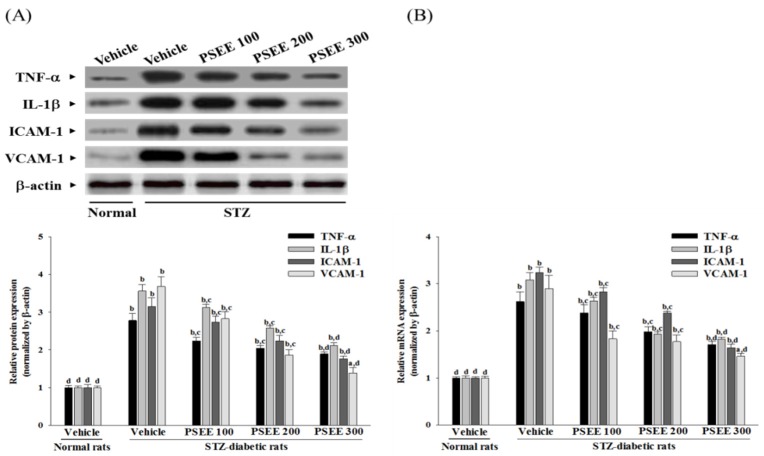
Changes in protein (**A**) and (**B**) mRNA levels of inflammatory cytokines and chemokines in retina of rats receiving 8-week treatments. STZ-diabetic rats (STZ) were dosed by oral gavage once per day for 8 weeks with PSEE at dosages of 100 (PSEE 100), 200 (PSEE 200), or 300 mg/kg (PSEE 300). Normal or STZ-diabetic rats receiving vehicle treatment were given the same volume of vehicle (distilled water) used to prepare the test medication solutions. Results in each column are mean ± SD from ten rats per group. ^a^ *p* < 0.05 and ^b^ *p* < 0.01 compared to vehicle-treated normal rats, respectively. ^c^ *p* < 0.05 and ^d^ *p* < 0.01 compared to the values of vehicle-treated STZ-diabetic rats, respectively.

In STZ-diabetic rats, the retinal protein levels of TNF-α, IL-1β, ICAM-1 and VCAM-1 were significantly increased by around 2.7-, 3.6-, 3.1- and 3.6-fold, respectively, as compared to those seen in the normal group (Figure 3A). Giving STZ-diabetic rats 300 mg/kg/day PSEE for 8 weeks markedly suppressed the retinal protein levels of TNF-α, IL-1β, ICAM-1 and VCAM-1 to 67.9%, 59.6%, 55.8% and 37.8% relative to those seen in the vehicle-treated counterparts, respectively (Figure 3A).

STZ caused a 2.6-fold increase in retinal TNF-α mRNA, a 3.1-fold rise in retinal IL-1β mRNA, a 3.2-fold increase in retinal ICAM-1 mRNA and a 2.9-fold rise in retinal VCAM-1 mRNA compared to the levels seen in the normal group (Figure 3B). Treatment with PSEE at the daily oral dosage of 300 mg/kg markedly suppressed the STZ-induced stimulation of retinal mRNA levels of TNF-α, IL-1β, ICAM-1 and VCAM-1 to 65.2%, 58.4%, 50.6% and 50.3%, respectively, compared to the levels seen in their vehicle-treated counterparts (Figure 3B). A positive correlation coefficient of 0.503 (*p* < 0.001), 0.412 (*p* < 0.001), 0.529 (*p* < 0.001), and 0.318 (*p* < 0.001), were identified between mRNA and protein expression in TNF-α, IL-1β, ICAM-1 and VCAM-1, respectively.

### 3.7. Effects on Retinal Angiogenic Factors Expression

**Figure 4:**
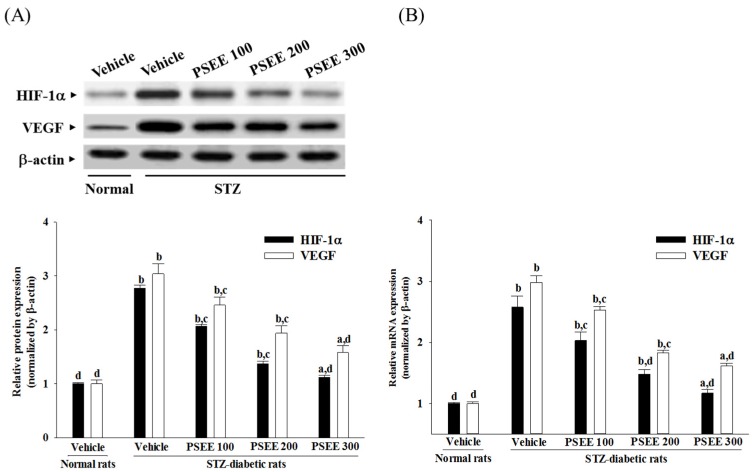
Changes in protein (**A**) and (**B**) mRNA levels of retinal angiogenic factors in retina of rats receiving 8-week treatments. STZ-diabetic rats (STZ) were dosed by oral gavage once per day for 8 weeks with PSEE at dosages of 100 (PSEE 100), 200 (PSEE 200), or 300 mg/kg (PSEE 300). Normal or STZ-diabetic rats receiving vehicle treatment were given the same volume of vehicle (distilled water) used to prepare the test medication solutions. Results in each column are mean ± SD from ten rats per group. ^a^ *p* < 0.05 and ^b^ *p* < 0.01 compared to vehicle-treated normal rats, respectively. ^c^ *p* < 0.05 and ^d^ *p* < 0.01 compared to the values of vehicle-treated STZ-diabetic rats, respectively.

Retinal protein and mRNA levels of HIF-1α in STZ-diabetic rats were clearly higher than those of the normal rats, and were down-regulated by 300 mg/kg/day PSEE treatment: decreases of 40.4% and 45.3%, respectively, when compared with the levels observed in the vehicle-treated counterparts (Figure 4).

The retinal expression of VEGF was significantly increased in the STZ-diabetic rats compared with those in normal rats at both protein and mRNA levels, which were decreased by 300 mg/kg/day PSEE treatment to 45.3% and 54.3%, respectively, relative to those observed in the vehicle-treated counterparts (Figure 4).

### 3.8. Effects on Protein Expression and Phosphorylation of Akt in Retinas

**Figure 5:**
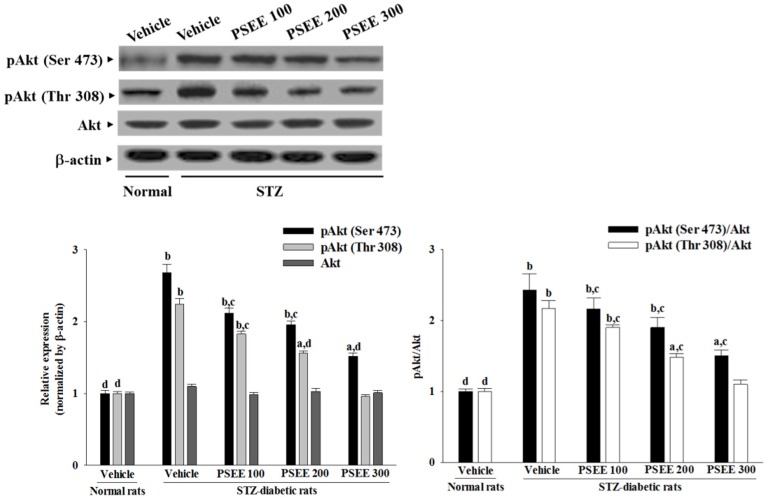
Effects of treatments on protein expression and phosphorylation of Akt in retinas. Representative immunoblots of retinal protein levels and phosphorylation degrees of Akt in rats receiving 8-week treatment. STZ-diabetic rats (STZ) were dosed by oral gavage once per day for 8 weeks with PSEE at dosages of 100 (PSEE 100), 200 (PSEE 200), or 300 mg/kg (PSEE 300). Normal or STZ-diabetic rats receiving vehicle treatment were given the same volume of vehicle (distilled water) used to prepare the test medication solutions. The pAkt/Akt ratio is expressed as the mean with mean ± SD from ten rats per group. ^a^ *p* < 0.05 and ^b^ *p* < 0.01 compared to vehicle-treated normal rats, respectively. ^c^ *p* < 0.05 and ^d^ *p* < 0.01 compared to the values of vehicle-treated STZ-diabetic rats, respectively.

No change was observed in the protein level of Akt in the retinas of STZ-diabetic rats compared with the normal group (Figure 5). PSEE did not affect the retinal Akt protein expression in STZ-diabetic rats (Figure 5). The immunoblot results showed that the phosphorylation of Akt on Ser 473 and Thr 308 were 2.6- and 2.2-fold greater in the retinas of STZ-diabetic rats than in the normal group, respectively (Figure 5). These STZ-induced upregulation in Akt phosphorylation was significantly reversed in the retinas after 8-week treatment with 300 mg/kg/day PSEE (56.6% decreases in Ser 473 and 57.1% decreases in Thr 308, relative to those in vehicle-treated STZ-diabetic rats; Figure 5). The STZ markedly elevated the ratio of pAkt (Ser 473)/Akt and pAkt (Thr 308)/Akt by 2.4- and 2.2-fold relative to those in vehicle-treated STZ-diabetic rats, respectively, in the retinas of the rats (Figure 5). Treatment of STZ-diabetic rats with 300 mg/kg/day PSEE significantly downregulated the ratios of pAkt (Ser 473)/Akt and pAkt (Thr 308)/Akt in the retinas to 1.5- and 1.1-fold relative to those in vehicle-treated STZ-diabetic rats (Figure 5).

### 3.9. Effects on Activities of NF-κB, HDAC and HAT in Retinas

**Figure 6:**
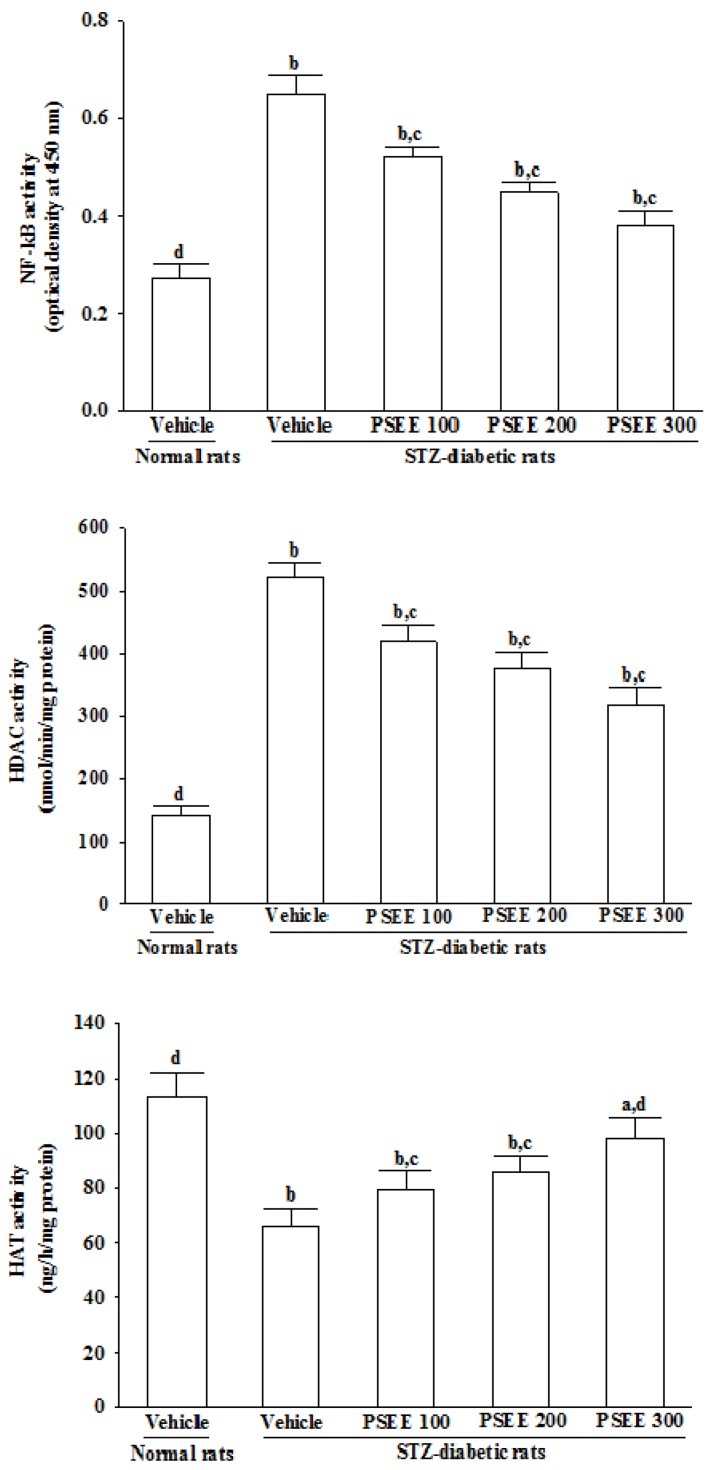
Activity of nuclear factor-κB (NF-κB), histone deacetylases (HDAC) and histone acetyltransferases (HAT) in retinas of rats receiving 8-week treatment. STZ-diabetic rats (STZ) were dosed by oral gavage once per day for 8 weeks with PSEE at dosages of 100 (PSEE 100), 200 (PSEE 200), or 300 mg/kg (PSEE 300). Normal or STZ-diabetic rats receiving vehicle treatment were given the same volume of vehicle (distilled water) used to prepare the test medication solutions. Results in each column are mean ± SD from ten rats per group. ^a^ *p* < 0.05 and ^b^ *p* < 0.01 compared to vehicle-treated normal rats, respectively. ^c^ *p* < 0.05 and ^d^ *p* < 0.01 compared to the values of vehicle-treated STZ-diabetic rats, respectively.

The activities of NF-κB and HDAC in the retinas of STZ-diabetic rats were 2.4- and 3.6-fold higher than those of the normal group, respectively (Figure 6). These STZ-induced up-regulations in activities of NF-κB and HDAC were lowered in the retina after treatment with 300 mg/kg/day PSEE, at 58.4% and 60.8% the levels seen in the vehicle-treated STZ-diabetic rats, respectively (Figure 6).

The retinal HAT activity was 58.6% lower in STZ-diabetic rats than in the normal group, which were enhanced by 300 mg/kg/day PSEE treatment, with a 1.6-fold elevation, respectively, when compared with the levels observed in the vehicle-treated counterparts (Figure 6).

## 4. Discussion

Data from multicenter prospective studies have shown that an abnormally high glucose concentration in blood is the principal cause of microvascular and macrovascular complications [1]. Therefore, tight control of blood glucose is the key to preventing or reversing diabetic complications in diabetic patients [11]. In vitro and in vivo studies have shown that dietary polyphenols may inhibit α-amylase and α-glucosidase, inhibit glucose absorption in the intestine by sodium-dependent glucose transporter 1, stimulate insulin secretion and reduce hepatic glucose output, suggesting that polyphenols could improve glucose homeostasis through potential multiple mechanisms of action and might be one dietary therapy for the prevention and management of diabetes [13]. In the present study, PSEE treatment showed significant and consistent reduction in fasting blood glucose levels and also improved the body weight loss in STZ-diabetic rats as compared to the vehicle treated diabetic controls, indicating its potent antidiabetic activity on an insulin deficient animal model. PSEE was rich in polyphenolic flavonoids, and its antidiabetic activity may be attributed to the presence of these.

Antioxidant nutrients and phytonutrients have been reported to inhibit the oxidation of living cells by free radicals and result in a decrease in oxidative stress [30]. In the present study, the antioxidant capacity of PSEE was measured by ABTS radical cation decolorization assay and showing promising results. It is clear that PSEE was rich in polyphenols and shown with antioxidant potential. Actually, long-term hyperglycemia could lead to an increase in ROS generation and decreased antioxidant capacity in diabetes [3]. The retina is particularly susceptible to oxidative stress because of high energy demands and exposure to light [31]. Regarding the oxidative stress affects the pathogenesis of DR, correction of oxidant-antioxidant balance may be a powerful approach for preventing vision loss associated with DR [3]. It is well known that SOD, CAT, and GSH constitute a mutually supportive team of defense against ROS [4]. In our study, decline in the activities of these enzymes in the retinal tissue of STZ-diabetic rats and attainment of near normalcy in PSEE-treated rats indicate that oxidative stress elicited in the retina of diabetic rats had been nullified due to the effect of PSEE. Thus, our results suggest that PSEE has potential to overcome the hyperglycemia-specific microvascular complications.

The growing evidence has suggested that BRB breakdown, leakage capillaries and vascular structural and functional changes are characteristic for the diabetic retina [6]. Similar with the previous study [32], we observed that the number of leukocytes adhered to the retinal vascular endothelium was increased in STZ-diabetic rats; accordingly, vascular permeability and the retinal vessels diameter were increased as well. In consistent with the attenuated leukostasis, PSEE reduced diabetic retinal vascular leakage accompanied by restrained the retinal vascular dilation in STZ-diabetic rats. Prevention of diabetes-related structural disorganization of the retina might be an important contributor of PSEE to the preventing the progression of DR.

VEGF, an endothelial angiogenic and vasopermeability factor, is known to be a key molecule leading to retinal permeability and breakdown of BRB in diabetes and other retinal diseases [33]. Regulation of VEGF expression is complex, and HIF-1α is one of the transcription factors that regulate VEGF expression under hyperglycemia [8,9]. Furthermore, Akt activation has been recognized as an upstream regulator of HIF-1α expression [34]. Therefore, promotion of the Akt-HIF-1α-VEGF signaling pathway contributes to the induction of retinal vascularization [35]. We found that the elevated contents of HIF-1α and VEGF in retinae of STZ-diabetic rats were both reduced in rats receiving PSEE treatment. In addition, the results of the present study revealed an increase in phosphorylation of Thr 308 and Ser 473 of Akt in the retinas of STZ-diabetic rats; the deficit was ameliorated by PSEE treatment. Therefore, it can be considered that PSEE rescued diabetic retinal vasculopathy by downregulation of HIF-1-mediated induction of VEGF expression via suppressing Akt activation in the retina of STZ-diabetic rats. Full activation of Akt requires phosphorylation on Thr 308 and Ser 473 by 3-phosphoinositide-dependent kinase-1 and Ser-473 kinase, respectively [36]. Thus, the role of PSEE on the alterations in Akt signaling in the development of DR will be identified in future research work.

Numerous studies show that hyperglycemia leads to oxidative stress in the diabetic retinas, which has been associated with cellular inflammation and release of inflammatory cytokines [2]. One of these mediators is TNF-α, a proinflammatory cytokine which is known as an initiator of inflammatory reactions [7]. Similarly, IL-1β can be up-regulated in the retina in diabetes [5]. In addition to increases in the above-mentioned inflammatory mediators, both molecules ICAM-1 and VCAM-1 promote chemoattraction of leukocytes into the vascular walls and their migration into retinal tissues, which accounts for the majority of diabetes-associated retinal vascular leakage [37]. Actually, plantaginis semen significantly inhibited lipopolysaccharide-induced cyclooxygenase-2 (concentration required for 50% inhibition [IC(50)] = 8.61 μg/mL, TNF-α [IC(50)] = 9.63 μg/mL, and nitric oxide [IC(50)] = 8.65 μg/mL) production in RAW 264.7 cells; anti-inflammatory activity of plantaginis semen has been reported [38]. In the present study, retinae from PSEE-treated STZ-diabetic rats showed lower levels of inflammatory cytokines and chemokines, suggesting that the extract acted against inflammatory response triggered by hyperglycemia. These results support the proposition that protection of PSEE from retinal damage in STZ-diabetic rats was mediated by blockade of diabetes-induced production of inflammatory molecules in retinal tissue, and attenuates retinal vascular leakage.

NF-κB plays a critical role in diabetes complications as it regulates transcription of a number of genes involved in inflammatory response [39]. Actually, it has been demonstrated that the subunits of the NF-κB signaling pathway, including the inhibitory transcription factor IκB, are acetylated/deacetylated by the HAT and HDAC, respectively [40]. High HDAC activity may therefore maintain deacetylated such inhibitory factor allowing for NF-κB activation [41]. Recent studies have shown that diabetes induced increase HDAC activity in the retina and kidney, that are the tissues associated with microvascular complications [42]. It has also been reported that hyperglycemia-induced superoxide overproduction activates HDAC activity and decreases HAT activity [43]. PSEE treatment results in a significant increase in HAT activity and a parallel decrease in activies of HDAC and NF-κB in retinae of STZ-diabetic rats. The effects of PSEE seems to play a role in controlling NF-κB activation and modulation of HDAC and HAT activity, consequently affecting the expression of inflammatory response genes.

Medicinal plants produced several useful biological activities; however, the inclusion of toxicological evaluation at preclinical stage will assure its safe usage in humans as a medicine [44]. Further studies are needed to clarify toxicity of PSEE to rat at the effective dosage used for treating DR. Whether PSEE is effective in human for DR improvement also need further evaluate in clinical studies.

## 5. Conclusions

The overall findings indicate that PSEE has protective effect on DR with possible mechanisms of lowering plasma glucose, rescuing oxidative stress and supression of angiogenesis via down-regulation of the HIF-1α/VEGF signaling axis accompanied by Akt inhibition. Impairment of NF-κB activation and maintain the balance between HAT and HDAC, and thereby limiting the inflammatory response has also been suggested as a possible underlying mechanism of PSEE involved in preventing the progression of diabetic retinal vascular diseases. PSEE supplementation may be considered as an alternative choice used for the prevention of retinal microvascular complications of diabetes. PSEE has abundant polyphenolic compounds with strong antioxidant activity. Polyphenols are divided into flavonoids, phenolic acids, stilbenes, and lignans [12]. Thus, the specific components of PSEE that are mainly responsible for its protective effects on retinas will be identified in future research work.

## Figures and Tables

**Table 1:** Changes in body weight, plasma levels of glucose and glycosylated hemoglobin as well as retinal antioxidant parameters in normal or streptozotocin (STZ)-diabetic rats receiving 8-week treatments.

Parameters	Normal Rats	STZ-Diabetic Rats			
	Vehicle	Vehicle	PSEE 100	PSEE 200	PSEE 300
Body weight (g/rat)	353.6 ± 10.3 ^d^	181.7 ± 8.9 ^b^	208.3 ± 9.3 ^b,c^	228.7 ± 11.3 ^b,c^	239.6 ± 10.7 ^b,c^
Plasma glucose (mg/dL)	93.5 ± 5.3 ^d^	432.1 ± 7.4 ^b^	398.5 ± 8.7 ^b,c^	341.5 ± 9.8 ^b,c^	288.9 ± 8.1 ^b,c^
HbA1c (%)	4.7 ± 0.8 ^d^	14.3 ± 1.2 ^b^	13.4 ± 1.1 ^b^	11.8 ± 0.9 ^b,c^	10.3 ± 1.0 ^b,c^
Retinal SOD (IU/mg protein)	8.4 ± 0.3 ^d^	2.7 ± 0.6 ^b^	4.1 ± 0.5 ^b,c^	5.6 ± 0.9 ^a,c^	6.9 ± 0.7 ^a,d^
Retinal CAT (IU/mg protein)	11.4 ± 1.2 ^d^	3.8 ± 0.3 ^b^	6.3 ± 0.5 ^a,c^	7.2 ± 0.7 ^a,d^	8.3 ± 0.6 ^a,d^
Retinal GSH (nmol/mg protein)	18.3 ± 2.7 ^d^	5.1 ± 1.4 ^b^	6.8 ± 2.1 ^b^	9.5 ± 1.8 ^b,c^	12.6 ± 2.3 ^a,d^

STZ-diabetic rats were dosed by oral gavage once per day for 8 weeks with PSEE at dosages of 100 (PSEE 100), 200 (PSEE 200), or 300 mg/kg (PSEE 300). Normal or STZ-diabetic rats receiving vehicle treatment were given the same volume of vehicle (distilled water) used to prepare the test medication solutions. Values (mean ± SD) were obtained for each group of ten animals. ^a^ *p* < 0.05 and ^b^ *p* < 0.01 compared to the values of vehicle-treated normal rats, respectively. ^c^ *p* < 0.05 and ^d^ *p* < 0.01 compared to the values of vehicle-treated STZ-diabetic rats, respectively.

## References

[1] Litwak L., Goh S.Y., Hussein Z., Malek R., Prusty V., Khamseh M.E. (2013). Prevalence of diabetes complications in people with type 2 diabetes mellitus and its association with baseline characteristics in the multinational A1chieve study. Diabetol. Metab. Syndr..

[2] El-Asrar A.M. (2012). Role of inflammation in the pathogenesis of diabetic retinopathy. Middle East Afr. J. Ophthalmol..

[3] Rochette L., Zeller M., Cottin Y., Vergely C. (2014). Diabetes, oxidative stress and therapeutic strategies. Biochim. Biophys. Acta.

[4] Rani V., Deep G., Singh R.K., Palle K., Yadav U.C. (2016). Oxidative stress and metabolic disorders: Pathogenesis and therapeutic strategies. Life Sci..

[5] Zhou J., Wang S., Xia X. (2012). Role of intravitreal inflammatory cytokines and angiogenic factors in proliferative diabetic retinopathy. Curr. Eye Res..

[6] Zhang C., Wang H., Nie J., Wang F. (2014). Protective factors in diabetic retinopathy: Focus on blood-retinal barrier. Discov. Med..

[7] Sharma S., Purohit S., Sharma A., Hopkins D., Steed L., Bode B., Anderson S.W., Caldwell R., She J.X. (2015). Elevated serum levels of soluble TNF receptors and adhesion molecules are associated with diabetic retinopathy in patients with Type-1 diabetes. Mediators Inflamm..

[8] Qaum T., Xu Q., Joussen A.M., Clemens M.W., Qin W., Miyamoto K., Hassessian H., Wiegand S.J., Rudge J., Yancopoulos G.D. (2001). VEGF-initiated blood-retinal barrier breakdown in early diabetes. Investig. Ophthalmol. Vis. Sci..

[9] Yan H.T., Su G.F. (2014). Expression and significance of HIF-1α and VEGF in rats with diabetic retinopathy. Asian Pac. J. Trop. Med..

[10] Malek M., Khamseh M.E., Aghili R., Emami Z., Najafi L., Baradaran H.R. (2012). Medical management of diabetic retinopathy: An overview. Arch. Iran. Med..

[11] Ostri C., la Cour M., Lund-Andersen H. (2014). Diabetic vitrectomy in a large type 1 diabetes patient population: Long-term incidence and risk factors. Acta Ophthalmol..

[12] Pandey K.B., Rizvi S.I. (2009). Plant polyphenols as dietary antioxidants in human health and disease. Oxid. Med. Cell. Longev..

[13] Kim Y., Keogh J.B., Clifton P.M. (2016). Polyphenols and glycemic control. Nutrients.

[14] Kang T., Zheng T., Jiang Y., Zhang X., Feng X. (1996). Commodity identification of semen Plantaginis and herba Plantaginis. China J. Chin. Mater. Med..

[15] Liu X., Wu X., Huang H., Zhong S., Lai X., Cao L. (2002). Herbalogical study on *Plantago asiatica* L.. China J. Chin. Mater. Med..

[16] Chen C.F., Ho W.T., Liao J.F., Chen S.M., Chow S.Y. (1980). Pharmacological studies of Chinese herbs. (8) Pharmacological effects of Plantaginis Semen (author’s transl). J. Formos. Med. Assoc..

[17] Samuelsen A.B. (2000). The traditional uses, chemical constituents and biological activities of *Plantago major* L. A review. J. Ethnopharmacol..

[18] Frati Munari A.C., Benítez Pinto W., Raúl Ariza Andraca C., Casarrubias M. (1998). Lowering glycemic index of food by acarbose and *Plantago psyllium* mucilage. Arch. Med. Res..

[19] Zhou Q., Lu W., Niu Y., Liu J., Zhang X., Gao B., Akoh C.C., Shi H., Yu L.L. (2013). Identification and quantification of phytochemical composition and anti-inflammatory, cellular antioxidant, and radical scavenging activities of 12 Plantago species. J. Agric. Food Chem..

[20] Majumdar S., Srirangam R. (2010). Potential of the bioflavonoids in the prevention/treatment of ocular disorders. J. Pharm. Pharmacol..

[21] Cai X., McGinnis J.F. (2016). Diabetic retinopathy: Animal models, therapies, and perspectives. J. Diabetes Res..

[22] Dóka O., Bicanic D. (2002). Determination of total polyphenolic content in red wines by means of the combined He-Ne laser optothermal window and Folin-Ciocalteu colorimetry assay. Anal. Chem..

[23] Djeridane A., Yousfi M., Nadjemi B., Boutassouna D., Stocker P., Vidal N. (2006). Antioxidant activity of some Algerian medicinal plants extracts containing phenolic compounds. Food Chem..

[24] Re R., Pellegrini N., Proteggente A., Pannala A., Yang M., Rice-Evans C. (1999). Antioxidant activity applying an improved ABTS radical cation decolorization assay. Free Radic. Biol. Med..

[25] Oto G., Ekin S., Ozdemir H., Demir H., Yasar S., Levent A., Berber I., Kaki B. (2011). Plantago major protective effects on antioxidant status after administration of 7,12-Dimethylbenz(a)anthracene in rats. Asian Pac. J. Cancer Prev..

[26] Gupta S.K., Kumar B., Nag T.C., Agrawal S.S., Agrawal R., Agrawal P., Saxena R., Srivastava S. (2011). Curcumin prevents experimental diabetic retinopathy in rats through its hypoglycemic, antioxidant, and anti-inflammatory mechanisms. J. Ocul. Pharmacol. Ther..

[27] Barber A.J., Antonetti D.A. (2003). Mapping the blood vessels with paracellular permeability in the retinas of diabetic rats. Investig. Ophthalmol. Vis. Sci..

[28] Xu Q., Qaum T., Adamis A.P. (2001). Sensitive blood-retinal barrier breakdown quantitation using Evans blue. Investig. Ophthalmol. Vis. Sci..

[29] Livak K.J., Schmittgen T.D. (2001). Analysis of relative gene expression data using real-time quantitative PCR and the 2−ΔΔCT method. Methods.

[30] Halliwell B., Gutteridge J.M., Cross C.E. (1992). Free radicals, antioxidants, and human disease: Where are we now?. J. Lab. Clin. Med..

[31] Kumari S., Panda S., Mangaraj M., Mandal M.K., Mahapatra P.C. (2008). Plasma MDA and antioxidant vitamins in diabetic retinopathy. Indian J. Clin. Biochem..

[32] Pouliot M., Talbot S., Sénécal J., Dotigny F., Vaucher E., Couture R. (2012). Ocular application of the kinin B1 receptor antagonist LF22-0542 inhibits retinal inflammation and oxidative stress in streptozotocin-diabetic rats. PLoS ONE.

[33] Fogli S., Mogavero S., Egan C.G., Del Re M., Danesi R. (2016). Pathophysiology and pharmacological targets of VEGF in diabetic macular edema. Pharmacol. Res..

[34] Lee B.L., Kim W.H., Jung J., Cho S.J., Park J.W., Kim J., Chung H.Y., Chang M.S., Nam S.Y. (2008). A hypoxia-independent up-regulation of hypoxia-inducible factor-1 by AKT contributes to angio-genesis in human gastric cancer. Carcinogenesis.

[35] Kim Y.G., Lim H.H., Lee S.H., Shin M.S., Kim C.J., Yang H.J. (2015). Betaine inhibits vascularization via suppression of Akt in the retinas of streptozotocin-induced hyperglycemic rats. Mol. Med. Rep..

[36] Bellacosa A., Chan T.O., Ahmed N.N., Datta K., Malstrom S., Stokoe D., McCormick F., Feng J., Tsichlis P. (1998). Akt activation by growth factors is a multiple-step process: The role of the PH domain. Oncogene.

[37] Khalfaoui T., Lizard G., Ouertani-Meddeb A. (2008). Adhesion molecules (ICAM-1 and VCAM-1) and diabetic retinopathy in type 2 diabetes. J. Mol. Histol..

[38] Kim B.H., Park K.S., Chang I.M. (2009). Elucidation of anti-inflammatory potencies of *Eucommia ulmoides* bark and *Plantago asiatica* seeds. J. Med. Food.

[39] Tak P.P., Firestein G.S. (2001). NF-kappaB: A key role in inflammatory diseases. J. Clin. Investig..

[40] Grabiec A.M., Tak P.P., Reedquist K.A. (2011). Function of histone deacetylase inhibitors in inflammation. Crit. Rev. Immunol..

[41] Ashburner B.P., Westerheide S.D., Baldwin A.S. (2001). The p65 (RelA) subunit of NF-kappaB interacts with the histone deacetylase (HDAC) corepressors HDAC1 and HDAC2 to negatively regulate gene expression. Mol. Cell. Biol..

[42] Lee H.B., Noh H., Seo J.Y., Yu M.R., Ha H. (2007). Histone deacetylase inhibitors: A novel class of therapeutic agents in diabetic nephropathy. Kidney Int. Suppl..

[43] Berthiaume M., Boufaied N., Moisan A., Gaudreau L. (2006). High levels of oxidative stress globally inhibit gene transcription and histone acetylation. DNA Cell Biol..

[44] Cuzzolin L., Zaffani S., Benoni G. (2006). Safety implications regarding use of phytomedicines. Eur. J. Clin. Pharmacol..

